# Associations Between Patient-Reported Outcome Measures of Physical and Psychological Functioning and Willingness to Share Social Media Data for Research Among Adolescents With a Chronic Rheumatic Disease: Cross-Sectional Survey

**DOI:** 10.2196/46555

**Published:** 2023-12-06

**Authors:** Elissa R Weitzman, Machiko Minegishi, Rachele Cox, Lauren E Wisk

**Affiliations:** 1 Division of Adolescent/Young Adult Medicine, Boston Children’s Hospital, Boston, MA, United States; 2 Department of Pediatrics, Harvard Medical School, Boston, MA, United States; 3 Division of Addiction Medicine, Boston Children’s Hospital, Boston, MA, United States; 4 Division of General Internal Medicine and Health Services Research, David Geffen School of Medicine, University of California Los Angeles, Los Angeles, CA, United States

**Keywords:** patient-reported outcomes, PROM, outcome measure, outcome measures, patient reported, patient data, social media, sharing, personally generated data, chronic illness, quality of life, rheumatic disease, rheumatic, rheumatoid, adolescent, adolescents, youth, research involvement, privacy, confidentiality, confidential, personal

## Abstract

**Background:**

Social media data may augment understanding of the disease and treatment experiences and quality of life of youth with chronic medical conditions. Little is known about the willingness to share social media data for health research among youth with chronic medical conditions and the differences in health status between sharing and nonsharing youth with chronic medical conditions.

**Objective:**

We aimed to evaluate the associations between patient-reported measures of disease symptoms and functioning and the willingness to share social media data.

**Methods:**

Between February 2018 and August 2019, during routine clinic visits, survey data about social media use and the willingness to share social media data (dependent variable) were collected from adolescents in a national rheumatic disease registry. Survey data were analyzed with patient-reported measures of disease symptoms and functioning and a clinical measure of disease activity, which were collected through a parent study. We used descriptive statistics and multivariate logistic regression to compare patient-reported outcomes between youth with chronic medical conditions who opted to share social media data and those who did not opt to share such data.

**Results:**

Among 112 youths, (age: mean 16.1, SD 1.6 y; female: n=72, 64.3%), 83 (74.1%) agreed to share social media data. Female participants were more likely to share (*P*=.04). In all, 49 (43.8%) and 28 (25%) participants viewed and posted about rheumatic disease, respectively. Compared to nonsharers, sharers reported lower mobility (T-score: mean 49.0, SD 9.4 vs mean 53.9, SD 8.9; *P*=.02) and more pain interference (T-score: mean 45.7, SD 8.8 vs mean 40.4, SD 8.0; *P*=.005), fatigue (T-score: mean 49.1, SD 11.0 vs mean 39.7, SD 9.7; *P*<.001), depression (T-score: mean 48.1, SD 8.9 vs mean 42.2, SD 8.4; *P*=.003), and anxiety (T-score: mean 45.2, SD 9.3 vs mean 38.5, SD 7.0; *P*<.001). In regression analyses adjusted for age, sex, study site, and Physician Global Assessment score, each 1-unit increase in symptoms was associated with greater odds of willingness to share social media data, for measures of pain interference (Adjusted Odds Ratio [AOR] 1.07, 95% CI 1.001-1.14), fatigue (AOR 1.08, 95% CI 1.03-1.13), depression (AOR 1.07, 95% CI 1.01-1.13), and anxiety (AOR 1.10, 95% CI 1.03-1.18).

**Conclusions:**

High percentages of youth with rheumatic diseases used and were willing to share their social media data for research. Sharers reported worse symptoms and functioning compared to those of nonsharers. Social media may offer a potent information source and engagement pathway for youth with rheumatic diseases, but differences between sharing and nonsharing youth merit consideration when designing studies and evaluating social media–derived findings.

## Introduction

Nearly 1 in 4 US youths are growing up with a chronic illness [1]. Many experience significant levels of disease and treatment burden (eg, pain and medication side effects) that undermine well-being, leading to frequent and costly health care utilization and family financial problems [2]. By adulthood, youth with a chronic illness face increased risks of poor educational, relationship, economic, and health outcomes [3,4]. Life-course risks reflect the complex interplay of disease and treatment experiences and the cumulative effects of social isolation, victimization, school disruption, psychological injury, and home life strain that can accompany chronic illness [5]. Capturing patients’ perspectives about these issues is vital to creating supportive interventions. This is especially true for adolescents, as the biopsychosocial processes of puberty, maturation, and development can impact the course and experience of chronic illness just as chronic illness experiences can impact these processes [6,7]. Patient-centered research with adolescents may advance understanding of these issues [8-10].

Psychosocial factors contribute to disease symptoms, such as pain and fatigue, among adolescents with a chronic illness [11]. However, we do not know, with regard to the day-to-day lives of adolescents, what issues are the most important to address to disrupt feedback between disease activity and psychosocial health [12-14]. For example, the experience of pain may be exacerbated by feelings of stress and isolation related to a chronic illness, which can be missed by clinicians when making a treatment decision. Discordance between a young patient’s disease experience, including their sense of well-being, and clinical manifestations of disease can stymie and misdirect treatment. This is important for chronic relapsing conditions that may have an unpredictable disease course with periods of flare and dormancy, such as pediatric-onset rheumatic diseases [15-18], which affect 1 in 250 US children younger than 18 years and account for an estimated US $8.3 billion in annual hospital charges [19,20]. Juvenile idiopathic arthritis (JIA) and systemic lupus erythematosus (SLE) are two common forms of pediatric-onset rheumatic disease. JIA is the most common cause of acquired disability in the United States and the fifth most common chronic childhood disease [21]. Youth with JIA report poorer health-related quality of life than that of their peers, even in the setting of low disease activity and after treatment with biologic agents [22-24]. The impacts of JIA persist into adulthood, by which time nearly half of affected youth still experience recurrent or ongoing disease activity, active arthritis, progressive joint destruction, and decreased health-related quality of life [22,25-28]. SLE is a lifelong, chronic, multisystem autoimmune disease; around 15% of persons with SLE developed it in childhood [29], and these persons typically experience severe phenotypes, including organ disease. Youth with SLE may experience secondary morbidities and psychosocial difficulties (eg, mood disorders, body image problems, and academic and social challenges [30]) because, in addition to life-threatening disease manifestations, treatment includes exposure to high doses and prolonged courses of glucocorticoids, as well as cytotoxic agents [31-33].

Patient-reported outcome (PRO) measures that capture dimensions of well-being can inform understanding of treatment experience and efficacy [33], and studies on the clinical validity of PROs are underway among youth with rheumatic diseases and other conditions [10,33]. Data gleaned from youths’ social media use may serve as an additional source of information about young patients’ experiences of disease and treatment.

Engaging youth with JIA and SLE in reporting about their health via social media and in sharing their social media data with investigators may provide a channel for learning about youth psychosocial status and physical functioning to complement clinical observations and PROs. Social media data may be obtained passively or actively. In the passive case, social media data may be obtained without youths’ permission or even without their awareness (as when data are programmatically collected and even sold by social media platforms). Additionally, social media data may be obtained actively via approaches that involve permissioned sharing, opt-in settings, and explicit notifications [34-38].

Regulatory efforts are being enacted to protect the privacy and autonomy of youth in web-based spaces [39,40]. As such, it is vital to understand whether young cohorts are willing to actively share their social media data for health research and whether health status differs between sharing and nonsharing groups [41]. Such insight would (1) clarify the feasibility of engaging youth with rheumatic diseases in sharing social media data for research and (2) help to quantify biases that could arise when relying on active models of collecting data from web-based cohorts. We sought to describe social media use among a clinically characterized cohort of youth with rheumatic diseases and to understand their passive (reading and viewing activities) and active (posting text or images) social media use in relation to these diseases. We further sought to quantify willingness to share social media data for health research and associations between willingness to share and patient-reported experiences of disease symptoms and functioning. We hypothesized that there would be equivalent percentages of youth who would and youth who would not agree to share their social media data for health research under a model of direct observation (ie, friending or otherwise providing access to social media data). Additionally, we hypothesized that the sharing cohort would report fewer symptoms and better functioning compared to those of the nonsharing cohort, potentially reflecting a greater sense of comfort and ease with their health and activities and fewer inhibitions about revealing any vulnerabilities. To date, few studies have been able to link personally generated data from social media with PROs and clinical data [42] to elucidate biases relevant to establishing the validity of social media data for health research—a recognized need [43,44].

## Methods

### Overview

Among adolescents with JIA or SLE who were members of the Childhood Arthritis and Rheumatology Research Alliance (CARRA) Registry [45,46] and enrolled in a prospective multisite study to clinically validate PRO measures [33], we investigated associations between social media use and willingness to share social media data for research. Survey reports were collected via a tablet computer by using the REDCap (Research Electronic Data Capture; Vanderbilt University) secure web application [47,48] at regularly scheduled clinics visits, during which PRO and clinical data were also collected.

### Ethical Considerations

A small stipend was provided to participants in the form of a US $20 gift card. Trained research assistants obtained in-person informed assent and assigned participants a unique study ID that was linked to the ID used for the clinical validation study and registry to ensure confidentiality. The study protocol was reviewed and approved by the Boston Children’s Hospital institutional review board (protocol number: IRB-P00025665).

### Setting and Sample

Adolescents were eligible if they were members of the CARRA Registry, were diagnosed with JIA or SLE, enrolled in the parent prospective clinical validation study [33], and were at 1 of 3 validation study sites that participated in this substudy. Additional eligibility criteria were an age of 13 to 18 years, the ability to complete the survey in English on a tablet computer, and the reported use of at least 1 of 4 popular social media platforms (Facebook, Twitter, Instagram, or Snapchat) in the past 30 days. Patients were ineligible if they were medically or emotionally unstable or were otherwise unable to assent, as determined by a clinician or site research team member; were unable to speak or read English at an eighth-grade reading level; or did not attend the data collection visit (absent at recruitment).

**Figure 1.**
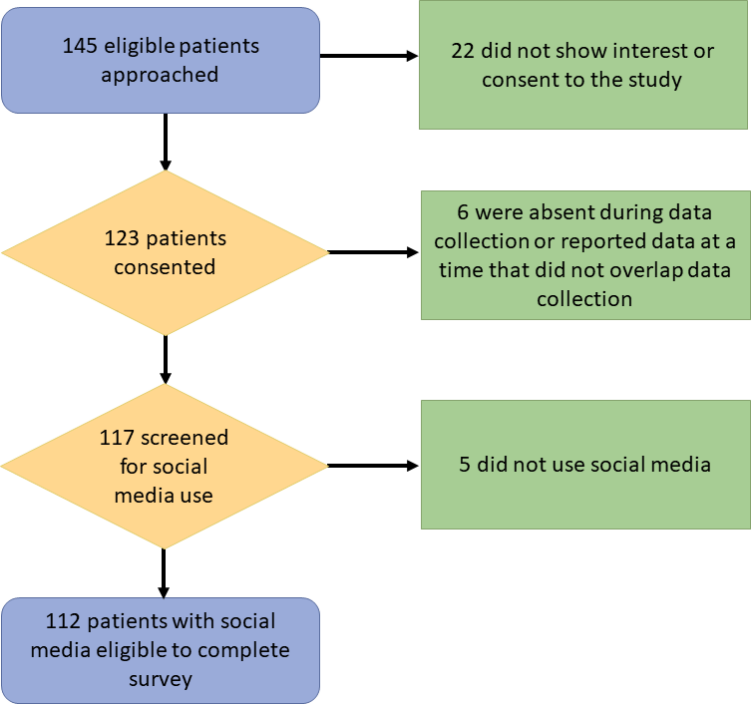
CONSORT (Consolidated Standards of Reporting Trials) diagram of study sample.

Of the 145 patients approached, 123 consented (84.8%), of whom 6 were excluded because they were absent during data collection or reported Patient-Reported Outcomes Measurement Information System (PROMIS) measures at a time that did not overlap social media data collection. Of the remaining 117 patients, 5 did not use Facebook, Twitter, Instagram, or Snapchat and were excluded, leaving an analytic sample of 112 (91.1%; Figure 1).

### Sources of Study Data and Measures

Clinical and demographic data were drawn from the CARRA Registry. PRO and social media survey data were collected electronically by using wireless touch screen tablets during the clinic visit. Social demographic measures included race, Hispanic ethnicity, sex at birth, date of birth, insurance status, and the highest education attained by a parent. Clinical characteristics included the study recruitment and treatment site, the Physician Global Assessment (PGA) score [49], a 10-point visual analog scale score (a value of ≥1 represented active disease), BMI, and disease duration in months.

PROMIS Pediatric measures [50,51] included short-form measures of fatigue, mobility, pain interference, depressive symptoms, anxiety, and meaning and purpose, which were administered by using computer-assisted technology [10,33]. Higher PROMIS symptom T-scores reflect worse symptom levels, and higher functioning scores reflect better functioning. PROMIS measures are designed such that the mean score of the relevant reference population (ie, healthy youth) is 50, with an SD of 10 [52]. A 3-point difference in the PROMIS Pediatric T-score metric is considered a minimally important difference [53].

Willingness to share social media data via direct observation by the research team was assessed for Facebook, Instagram, Twitter, and Snapchat. Willingness measures are summarized in Table 1, along with related measures of motivation to share social media data, reasons for not sharing, and passive and active use patterns.

**Table 1.** Measures of willingness to share social media data.

Question		Response options	Answers
**Willingness to share social media**			
	“Are you willing to share your social media posts from the following site(s) for the two-week time interval around this study visit and your next study visit? This involves ‘friending’ the study account so the study team can view your posts. We will not message, ‘like,’ post on, or interact with your accounts”	Facebook Twitter Instagram Snapchat	Yes No Prefer not to answer
	“(If YES to sharing any SM with the study) How much do you agree with the following statements regarding your motivations for sharing your social media data with this study?”	I will be able to help other patients with rheumatic conditions I am interested in research I am interested in social media and technology Participating in this research makes me feel valued The $20^a^ gift card incentivized me to participate in this research I have some other motivation for sharing my social media data with this study	Strongly agree Agree Disagree Strongly disagree Prefer not to answer
**Frequency of social media use**			
	“About how often do you visit OR use the following social media sites?”	Facebook Twitter Instagram Snapchat	Several times a day About once a day A few times a week Every few weeks Less often I do not use this site Prefer not to answer
	“How often do you VIEW/READ about other people who have a rheumatic condition on any of the following sites?”	Facebook Twitter Instagram Snapchat	Often Sometimes Rarely Never Prefer not to answer
	“Have you ever POSTED about your rheumatic condition on social media?”	N/A^b^	Yes No Prefer not to answer
**Benefits of viewing/reading about others with RD^c^**			
	“VIEWING/READING about other people who have a rheumatic condition on social media…”	Helps me to feel less alone with my rheumatic condition Helps me to talk to my friends about my rheumatic condition Helps me to feel more prepared when talking to my doctor/care team about my rheumatic condition Provides me with information about my condition that I can understand Helps me to talk to my parents or guardians about my rheumatic condition Provides me with information about treatments for my rheumatic condition Provides me with health information that my doctors/care team cannot provide Provides me with health information that I cannot find anywhere else	Strongly agree Agree Disagree Strongly disagree Prefer not to answer
**Motivations for posting**			
	“How much do the following reasons motivate you to POST/SHARE about your condition on social media?”	I want to feel understood I want to help or provide support to other people living with a rheumatic condition or any chronic health condition I want to connect with others living with a rheumatic condition or any chronic health condition I want to share my experiences with a community that believes me I want to update my friends/family members about my rheumatic condition I want to get help or support from others who are living with a rheumatic condition or any chronic health condition I want to share my thoughts/feelings when my rheumatic condition is under good control I want to share my thoughts/feelings when I am experiencing disease symptoms	A great deal Somewhat Very little Not at all Prefer not to answer
	“How important to you are the following reasons when you are making decisions NOT to POST/SHARE about your rheumatic condition?”	My rheumatic condition does not define me I do not want others to feel bad for me because of my rheumatic condition I do not want to disclose my diagnosis public on the internet My rheumatic condition is not serious enough for me to post about it on social media I worry about others knowing too much about my health I do not want my friends to find out how I am feeling or doing People might make fun of me, or I might get teased/bullied I do not want my parents or guardians to find out how I am feeling or doing	A great deal Somewhat Very little Not at all Prefer not to answer

a US $20.

b N/A: not applicable.

c RD: rheumatic disease.

### Data Analyses

Summary statistics were computed to characterize the study sample overall and by willingness to share social media data for research. The differences in demographic characteristics based on willingness to share social media data were analyzed by using appropriate statistical tests, including the Kruskal-Wallis test, 2-tailed *t* test, 2-sided Fisher exact test, and chi-square test. For 2 participants with JIA and 1 patient with SLE, we imputed missing values on the PGA by using the median score for their disease group and similarly imputed missing BMI values for 2 participants with JIA. In separate multivariable logistic regressions, we assessed the associations between willingness to share social media data for research (the dependent variable) and each PROMIS measure. All models controlled for age, biological sex, study site, and PGA score. The analyses were conducted by using SAS 9.4 (SAS Institute) [54]. Statistical significance was considered at *P*<.05.

## Results

### Sample Characteristics

Of the 112 participants, 98 (87.5%) were persons with JIA and 14 (12.5%) were persons with SLE. Overall, participants were aged 13 to 18 (mean 16.1, SD 1.6) years, 72 (64.3%) were female, 86 (76.8%) were White, 105 (93.8%) were non-Hispanic, and 101 (90.2%) had private insurance. For all, the average BMI was 23.2 (SD 4.3) kg/m^2^, and the average PGA score was 0.8 (SD 1.5), indicating inactive disease.

Willingness to share social media data was reported by a majority (83/112, 74.1%) of participants. In all, 43.8% (49/112) reported viewing or reading about others with rheumatic diseases on social media, and 25% (28/112) reported posting about rheumatic disease (Table 1).

### Association Between Willingness to Share Social Media Data and Health Status

Willingness to share social media data was associated with female sex (*P*=.04) and greater disease activity (*P*=.04), which was measured as a mean PGA score (Table 2). Compared to nonsharers, sharers reported lower mobility (T-score: mean 49.0, SD 9.4 vs mean 53.9, SD 8.9; *P*=.02), greater pain interference (T-score: mean 45.7, SD 8.8 vs mean 40.4, SD 8.0; *P*=.005), more fatigue (T-score: mean 49.1, SD 11.0 vs mean 39.7, SD 9.7; *P*<.001), more depression (T-score: mean 48.1, SD 8.9 vs mean 42.2, SD 8.4; *P*=.003), and greater anxiety (T-score: mean 45.2, SD 9.3 vs mean 38.5, SD 7.0; *P*<.001).

In logistic regression analyses that controlled for age, sex, study site, and PGA score, each 1-unit increase in symptoms was associated with greater odds of willingness to share social media data, for measures of pain interference (Adjusted Odds Ratio [AOR] 1.07, 95% CI 1.001-1.14), fatigue (AOR 1.08, 95% CI 1.03-1.13), depression (AOR 1.07, 95% CI 1.01-1.13), and anxiety (AOR 1.10, 95% CI 1.03-1.18; Table 3).

**Table 2.** Characteristics of the sample by willingness to share social media.

Characteristics			All participants (N=112)	Social media sharing		*P* value
				Yes (n=83, 74.1%)	No (n=29, 25.9%)	
Age (years), mean (SD)			16.1 (1.6)	16.2 (1.6)	15.9 (1.5)	.34
**Biological sex, n (%^a^)**						.04
	Male		40 (35.7)	25 (30.1)	15 (51.7)	
	Female		72 (64.3)	58 (69.9)	14 (48.3)	
**Race, n (%^a^)**						.51
	White		86 (76.7)	65 (78.3)	21 (72.4)	
	Asian		5 (4.5)	2 (2.4)	3 (10.3)	
	African American		2 (1.8)	2 (2.4)	0 (0)	
	Mixed race		4 (3.6)	3 (4.6)	1 (3.4)	
	Other race^b^		6 (5.4)	5 (6)	1 (3.4)	
	Unknown		9 (8)	6 (7.2)	3 (10.3)	
**Ethnicity, n (%^a^)**						.67
	Hispanic		7 (6.3)	6 (7.2)	1 (3.4)	
	Non-Hispanic		105 (93.8)	77 (92.8)	28 (96.6)	
**Parental education, n (%^a^)**						.10
	Less than a college degree		2 (1.8)	0 (0)	2 (6.9)	
	College degree or higher		39 (34.8)	29 (34.9)	10 (34.5)	
	Prefer not to answer or missing		71 (63.4)	54 (65.1)	17 (58.6)	
**Insurance, n (%^a^)**						.15
	Private health insurance		101 (90.2)	77 (92.8)	24 (82.8)	
	Government insurance or other		11 (9.8)	6 (7.2)	5 (17.2)	
**Rheumatic disease diagnosis, n (%^a^)**						.19
	Systemic lupus erythematosus		14 (12.5)	8 (9.6)	6 (20.6)	
	Juvenile idiopathic arthritis		98 (87.5)	75 (90.4)	23 (79.3)	
**Health characteristics, mean (SD)**						
	BMI (kg/m^2^)		23.2 (4.3)	23.3 (4.3)	22.7 (4.6)	.39
	Disease duration (months)		84.6 (53.8)	84.2 (52.9)	85.6 (57.1)	>.99
	Physician Global Assessment (score)		0.8 (1.5)	1.0 (1.6)	0.5 (1.1)	.04
**PROMIS**^**c**^ **Pediatric measure (T-score)**						
	Mobility		50.3 (9.5)	49.0 (9.4)	53.9 (8.9)	.02
	Pain interference		44.3 (8.9)	45.7 (8.8)	40.4 (8.0)	.005
	Fatigue		46.7 (11.4)	49.1 (11.0)	39.7 (9.7)	<.001
	Depressive symptoms		46.6 (9.1)	48.1 (8.9)	42.2 (8.4)	.003
	Anxiety		43.5 (9.2)	45.2 (9.3)	38.5 (7.0)	<.001
	Meaning and purpose^d^		47.4 (8.3)	47.4 (8.6)	47.2 (7.4)	.91
Passive social media use, n (%^a^)			49 (43.8)	39 (47)	10 (34.5)	.24
Active social media use, n (%^a^)			28 (25)	23 (27.7)	5 (17.2)	.26

a Column percentages are shown (ie, the percentages were calculated by using the n values presented in the “All Participants” [N=112], “Yes” [n=83], and “No” [n=29] headings as the denominators).

b Includes Middle Eastern or North African, Native American, American Indian, Alaska Native, Native Hawaiian, or other Pacific Islander.

c PROMIS: Patient-Reported Outcomes Measurement Information System.

d PROMIS measures for meaning and purpose were assessed among a total of 105 participants, including 80 (76.2%) in the “Yes” social media sharing group. Out of 112 total participants, 7 were not administered the PROMIS Meaning and Purpose survey during the parent study visit for reasons of timing.

**Table 3.** Associations between PROMIS^a^ Pediatric measures of physical functioning, symptoms, and psychosocial well-being and social media sharing (N=112).^b^

PROMIS Pediatric measure	Adjusted odds ratio^c^ (95% CI)	
Mobility	0.95 (0.89-1.003)	
Pain inference	1.07 (1.001-1.14)	
Fatigue	1.08 (1.03-1.13)	
Depressive symptoms	1.07 (1.01-1.13)	
Anxiety	1.10 (1.03-1.18)	
Meaning and purpose^d^	1.01 (0.95-1.07)	

a PROMIS: Patient-Reported Outcomes Measurement Information System.

b Outcome: sharing social media contents (reference: not sharing social media); exposure: 1-unit increase in the PROMIS.

c Adjusted models controlled for the participants’ age, sex, study sites, and Physician Global Assessment score. The reference group for the model is the participant group that was not willing to share the contents of any of their social media platforms with the researchers for this study. For each model, each individual PROMIS measure was entered independently, so these estimates do not reflect adjustment for other PROMIS measures.

d PROMIS measures for meaning and purpose were assessed among a total of 105 participants, including 80 (76.2%) in the “Yes” social media sharing group. Out of 112 total participants, 7 were not administered the PROMIS Meaning and Purpose survey during the parent study visit for reasons of timing.

### Social Media Use and Value for Youth With Rheumatic Disease

The use of Instagram, Snapchat, Facebook, and Twitter was reported by 94.6% (106/112), 83.9% (94/112), 42.9% (48/112), and 31.3% (35/112) of participants, respectively, and poly-platform use was reported by 84.8% (95/112) of participants. More than two-fifths of participants (49/112, 43.8%) reported passive social media use, that is, reading about others with rheumatic diseases, while one-quarter (28/112, 25%) reported active social media use, that is, posting about rheumatic disease. Passive and active use patterns did not differ by age or by diagnosis; however, larger percentages of female participants than male participants reported both passive and active rheumatic disease–related social media activity (Table 4).

**Table 4.** Sample characteristics by passive or active social media use.

Characteristics		All participants (N=112)	Read about others with RD^a^ on SM^b^			Post about RD on SM		
			Yes (n=49, 43.8%)	No (n=63, 56.3%)	*P* value	Yes (n=28, 25%)	No (n=84, 75%)	*P* value
Age (years), mean (SD)		16.1 (1.6)	16.3 (1.6)	16.0 (1.6)	.32	16.3 (1.6)	16.1 (1.6)	.61
**Biological sex, n (%)**								
	Male	40 (35.7^c^)	9 (22.5^d^)	31 (77.5^d^)	<.001	2 (5^d^)	38 (95^d^)	<.001
	Female	72 (64.3^c^)	40 (55.6^d^)	32 (44.4^d^)	N/A^e^	26 (36.1^d^)	46 (63.9^d^)	N/A
**Rheumatic disease diagnosis, n (%)**								
	Systemic lupus erythematosus	14 (12.5^c^)	9 (64.3^d^)	5 (35.7^d^)	.10	5 (35.7^d^)	9 (64.3^d^)	.33
	Juvenile idiopathic arthritis	98 (87.5^c^)	40 (40.8^d^)	58 (59.2^d^)	N/A	23 (23.5^d^)	75 (76.5^d^)	N/A
BMI (kg/m^2^), mean (SD)		23.2 (4.3)	23.3 (4.3)	23.0 (4.4)	.78	24.4 (4.8)	22.7 (4.1)	.12
Disease duration (months), mean (SD)		84.6 (53.8)	76.9 (49.2)	90.5 (56.7)	.21	83.2 (48.7)	85.0 (55.6)	.96
Physical Global Assessment (score), mean (SD)		0.8 (1.5)	0.8 (1.2)	0.8 (1.7)	.23	0.6 (0.7)	0.9 (1.6)	.99

a RD: rheumatic disease.

b SM: social media.

c This percentage was calculated by using the total number of participants (N=112) as the denominator.

d A row percentage is presented (ie, the n value in the corresponding “All Participants” row total was used as the denominator).

e N/A: not applicable.

**Figure 2.**
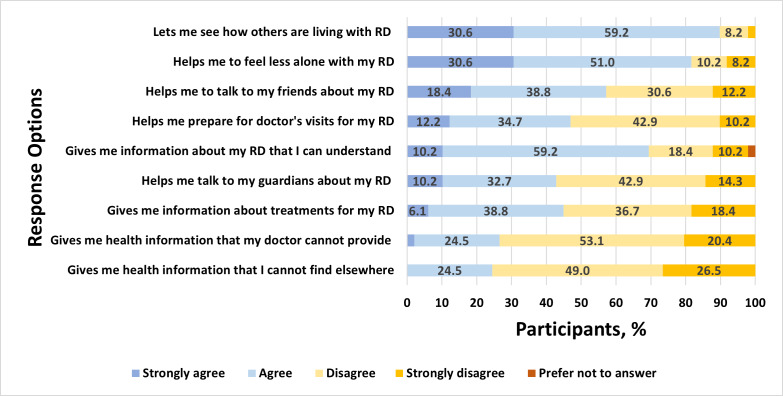
Responses for the following measure: “VIEWING/READING about other people who have a rheumatic condition on social media.” RD: rheumatic disease.

**Figure 3.**
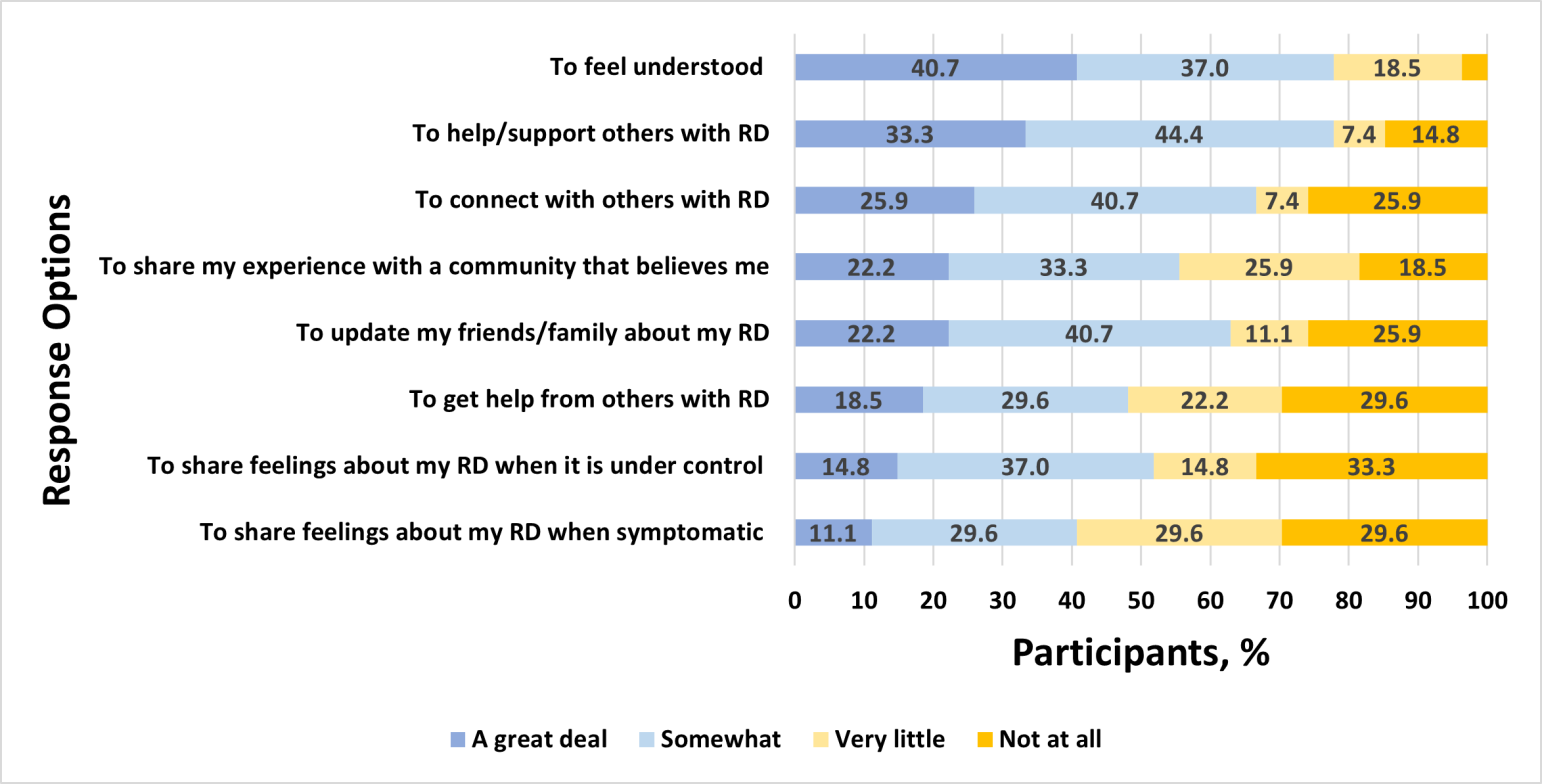
Responses for the following measure: “How much do the following reasons motivate you to POST/SHARE about your condition on social media?” RD: rheumatic disease.

**Figure 4.**
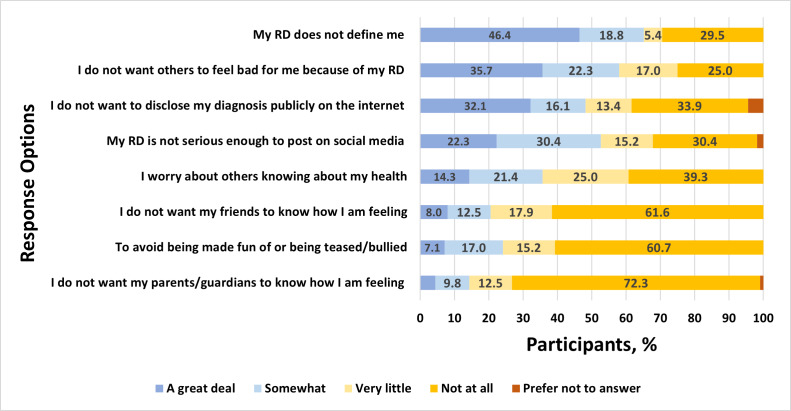
Responses to the following measure: “How important to you are the following reasons when you are making decisions NOT to POST/SHARE about your rheumatic condition?” RD: rheumatic disease.

**Figure 5.**
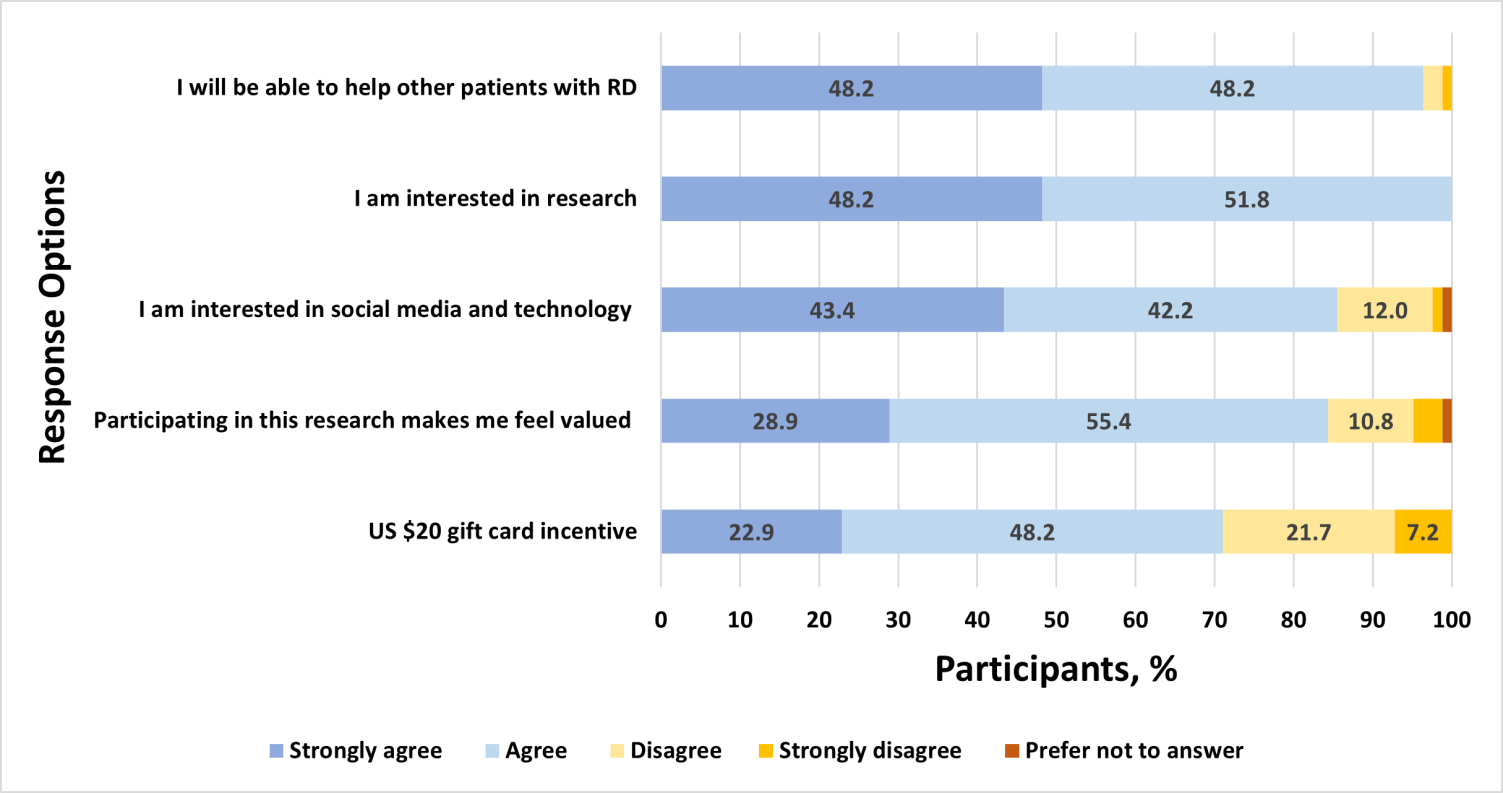
Responses to the following measure: “How much do you agree with the following statements regarding your motivations for sharing your social media data with this study?” RD: rheumatic disease.

Among 49 youths who reported passive disease-related use of social media, there were high levels of agreement that such use is helpful for observational learning from others and for alleviating feelings of isolation (Figure 2). Similarly, many reported that passive use increases their access to understandable information and helps them feel prepared in speaking with family and their care team about rheumatic disease. Of the 28 social media users who reported that they use social media actively to post about rheumatic disease, many reported doing so to feel understood, support and connect with others with rheumatic diseases, and share experiences with family and the rheumatic disease community (Figure 3). Among all social media users, a plurality endorsed the importance of not defining themselves by their condition and not wanting others to feel badly for them because of their condition, reporting these as reasons for *not* posting about their condition. Others refrained from posting about their condition to avoid public disclosure, retain privacy, and protect themselves from others’ attention or because they considered their condition insufficiently serious to merit attention (Figure 4). Finally, participants who shared their social media data reported they were motivated to do so because they were interested in research or technology, out of altruism toward other patients with the same conditions, to feel valued, and to receive a stipend (Figure 5).

## Discussion

### Principal Findings

In this multisite cohort study centered on adolescents with rheumatic diseases, we found high levels of willingness to share social media data for health research. Contrary to our original hypothesis that willingness to share would be associated with lower levels of symptoms and better psychosocial status, sharing was associated with higher levels of disease-related symptoms, specifically pain interference and fatigue, and higher levels of depression and anxiety. Both passive and active disease-related use of social media were reported, and larger percentages of female participants than male participants engaged in these activities. Passive and active social media use patterns were motivated by participants’ goals regarding observational learning about their condition and connection to others with similar conditions, as well as by the exchange of social support. Motivations to refrain from posting about rheumatic disease reflected participants’ goals of wanting to avoid having their condition define them, remaining private, and protecting against disclosure and ridicule. Among participants who agreed to share their social media data for research (n=83), almost all did so out of interest in research (83/83, 100%), to help others with a rheumatic condition (80/83, 96%), and because they felt it was of value personally (70/83, 84%) and financially (ie, for compensation; 59/83, 71%).

A growing body of research indicates the potential for improving adolescent and young adult health behaviors and outcomes (eg, health food consumption, reduced BMI, and reduced tobacco use) through engagement with social media and related features for peer groups and messaging [55-57]. Nevertheless, understanding of the potential for using social media as a source of health information and as a platform for research engagement is constrained by the lack of insight into the differences in health status between persons who are and persons who are not willing to share their data. For participatory surveillance models, some evidence shows that greater sharing and openness exist among early adopters of research apps and among persons whose disease is better controlled [58]. Other studies have found less reticence to share digital health data for care improvement among technology users who report lower incomes when compared to those who report higher incomes [59].

This study adds to what is known about willingness to share social media, with the added advantage of assessing reports from a clinically characterized cohort whose actual sharing was directly observed, differing from studies on hypothetical willingness, which are more common [60-62]. Few studies of social media use and social media data sharing provide access to linked clinical data [42] or structured PROs, and to our knowledge, none have been undertaken among youth with rheumatic diseases. Research with teenagers is vital, since this group is assuming control over their own health care, health information, and social media, and as a group, teenagers are both heavily engaged with social media [63] and uniquely vulnerable to social influences communicated through web-based channels [58,59,64,65].

Our findings of differences between sharing and nonsharing cohorts have implications for the use of personally generated data from web-based cohorts. In this study, sharing was associated with worse health. It is not clear what explains differences in health status between sharing and nonsharing youth. It may be that youth with rheumatic diseases who are unwilling to share their social media data are more socially engaged in ways that they consider unsuitable or too sensitive to allow sharing. Alternatively, they may be less desirous of research attention if they feel well and are able to satisfy their social needs through offline means. Future work may help elucidate reasons for observed differences. Similarly, future work with youth affected by other conditions is merited to understand whether differences between sharing and nonsharing groups hold.

The findings from this study have larger implications for social media–related research. First, investigators who use social media platforms to engage adolescents in health research should be explicit about the potential for biases related to inferences when denominators are poorly specified or are unknown [41,66]. The results from this study suggest that observations about the health of youth with rheumatic diseases drawn from social media–engaged cohorts may be skewed toward describing youth with greater symptoms and worse psychosocial health. This is a limitation when the goal of a study is to understand the entirety of a patient population but may be an advantage when the aim of a study is to engage youth who are struggling. The internet and social media serve important supportive functions for youth with a chronic illness, of whom many have been disproportionately adversely affected by social isolation and the hardships of the recent COVID-19 pandemic [67]. Second, findings regarding the high value placed by youth with rheumatic diseases on obtaining social and informational support related to their condition from others on the web is revealing of the seriousness of the gaps in support available from organized health care systems, as others have reported [68]. As youth turn to social media for support to fill these gaps, it remains important to consider the accuracy and safety of information offered by web-based peers, accessibility to youth across a range of health literacy and technology access levels, and the potential for harm from exposure to misinformation. Steps for detecting and addressing this during clinical visits might include asking young patients about their need for information and support and their ability to access web-based resources, as well as providing links and pointers to reliable, vetted sources of web-based guidance. These “low-tech” strategies can be implemented among even more computationally sophisticated systems for evaluating and improving the quality and accuracy of web-based information.

### Limitations

This report draws from a convenience sample of youth, and generalizability is limited by several factors, including the focus on youth with rheumatic diseases who have access to and use the internet and social media. The study cohort consisted of youth who were previously enrolled in research, and our findings may not generalize to youth who refrain from or have limited access to research opportunities. Sociodemographic diversity is also limited. However, the clinical confirmation of disease status and availability of validated health measures (eg, PROMIS measures) are strengths. Nevertheless, the results may not generalize to youth at other clinical sites, a broader sample of youth with rheumatic diseases, youth with other chronic conditions, or youth who do not use social media. This study did not assess participants’ understanding of health information or their ability to discern information quality or misinformation. The cross-sectional nature of this study precludes causal interpretation.

### Conclusions

We found high willingness to share social media data for health research among a clinically characterized cohort of adolescents with rheumatic diseases and substantial use of social media for disease-related observational learning and social connection. Differences in health status between sharing and nonsharing youth (as well as between social media users and nonusers) underscore the importance of considering the potential for biases in research results that rely on social media data and the importance of identifying opportunities to engage and improve the health of youth who may be on the web and in need of support.

## Abbreviations

AOR: adjusted odds ratio
CARRA: Childhood Arthritis and Rheumatology Research Alliance
JIA: Juvenile Idiopathic Arthritis
PGA: Physician Global Assessment
PRO: Patient-Reported Outcome
PROMIS: Patient-Reported Outcomes Measurement Information System
REDCap: Research Electronic Data Capture
SLE: systemic lupus erythematosus
